# A Novel Error Model of Optical Systems and an On-Orbit Calibration Method for Star Sensors

**DOI:** 10.3390/s151229863

**Published:** 2015-12-12

**Authors:** Shuang Wang, Yunhai Geng, Rongyu Jin

**Affiliations:** Research Center of Satellite Technology, Harbin Institute of Technology, Harbin 150001, China; wangshuang_girl@163.com (S.W.); amyjinrongyu@163.com (R.J.)

**Keywords:** star sensor, image plane rotary-tilt errors, distortions, two-step calibration method

## Abstract

In order to improve the on-orbit measurement accuracy of star sensors, the effects of image-plane rotary error, image-plane tilt error and distortions of optical systems resulting from the on-orbit thermal environment were studied in this paper. Since these issues will affect the precision of star image point positions, in this paper, a novel measurement error model based on the traditional error model is explored. Due to the orthonormal characteristics of image-plane rotary-tilt errors and the strong nonlinearity among these error parameters, it is difficult to calibrate all the parameters simultaneously. To solve this difficulty, for the new error model, a modified two-step calibration method based on the Extended Kalman Filter (EKF) and Least Square Methods (LSM) is presented. The former one is used to calibrate the main point drift, focal length error and distortions of optical systems while the latter estimates the image-plane rotary-tilt errors. With this calibration method, the precision of star image point position influenced by the above errors is greatly improved from 15.42% to 1.389%. Finally, the simulation results demonstrate that the presented measurement error model for star sensors has higher precision. Moreover, the proposed two-step method can effectively calibrate model error parameters, and the calibration precision of on-orbit star sensors is also improved obviously.

## 1. Introduction

The improvement of the attitude accuracy of satellites has stressed the demand for high-precision attitude instruments [1]. Star sensors are some of the most accurate attitude determination instruments [2], of which the precision affects the accuracy of satellite control systems directly, and depends highly on their optical parameters. However, these optical parameters generally provided by ground-based calibration are affected by several factors, such as the intensive vibration during the launching process, instrument aging, and the space environment [3,4,5]. In order to enhance the measurement accuracy of star sensors, it is desirable to investigate the problem of their on-orbit calibration and compensation [6].

Recently, the most widely-used error model of star sensors was merely to calibrate the principal point drift and the focal length error of optical systems [6,7,8,9]. However, the on-orbit space environment will inevitably lead to distortions, rotation and tilt of the optical systems [10,11]. That means, the distortions and the rotary-tilt errors of the image-plane also exist in the actual error model. Sun [12] analyzed the effects of image-plane displacement errors on star sensors’ measurement precision, and pointed out that the image-plane tilt error and rotary error decrease the measurement accuracy of the star sensors sharply. Sun [13] developed the Euler-axis-angle-based error model with six-degree-of-freedom image plane displacement errors, which considers the principal point drift, the focal length error and the tilt-rotary errors. However the vector property of the Euler axis has not been considered in this model. Moreover, most of the existing results on lens distortions are focused on the field of robot vision recognition systems and observation cameras [14,15]. The distortions of the optical systems are rarely mentioned in the recent studies on the calibration of high-precision star sensors. It has been proved that lens distortions have a great effect on the large field of view star sensors and the effect cannot be ignored with the development of star sensors [16]. In conclusion, establishing a comprehensive error model including the principal point drift , the focal length error, the distortions as well as the image plane tilt and rotary errors, is important for high-precision star sensors [17,18]. The effect of the image plane rotary-tilt errors and the distortions on measurement accuracy of star sensors is worthy of further investigation.

The existing on-orbit calibration methods mostly use the traditional error model. For example, Samaan *et al*. [19] developed a recursive Kalman filter for star sensor optical system calibration. In his approach, the least-squares estimate is adopted to determine the principal point offset and focal length. Then the results of the least-squares estimate are used as the “measurements” input for a recursive Kalman filter to filter out the noise. Liu *et al*. [10] proposed a modified version of the least-squares iteration algorithm for autonomous on-orbit calibration of the star sensor camera. Firstly, the optimal principal point and focal length are obtained, and then the high-order focal-plane distortions are estimated by using the solution of the first step. However, for the distortions, the image plane tilt error and the rotary error, the above calibration methods are inapplicable. They cannot estimate the error parameters directly due to the existence of strong nonlinearity among these parameters. The Extended Kalman Filter (EKF) is a typical parameter estimation method, which has excellent filtering capability in nonlinear systems with white noise [20,21,22]. However, the EKF cannot directly obtain the estimates of the image plane rotary-tilt error angles, because the image plane rotary-tilt errors are nonlinear, and the corresponding attitude transformation matrixes are orthonormal.

Based on the above discussions, in this paper, a novel star sensor on-orbit calibration error model is suggested, which considers the principal point drift, the focal length error, the image plane tilt and rotary errors, and the lens distortions within a unified framework. Compared with the traditional error model, the new model is more comprehensive and suitable for high-precision star sensors. The impact of these errors on the accuracy of star sensors is analyzed systematically. Additionally, we are inspired by the use of two star sensors for calibration in the Gravity Recovery and Climate Experiment (GRACE) and the Gravity Field and Ocean Circulation Explorer (GOCE), which has a significantly contribution on the accuracy of satellite gravimetry [23,24,25,26]. Therefore, an improved two-step calibration method, which is based on the EKF and Least Square Method (LSM) and takes advantage of two star sensors, is proposed to calibrate the above errors on-orbit. The designed method is employed to estimate the corresponding error parameters step by step. The EKF is used to calibrate the main point drift, the focal length error and the distortions of the optical systems, while the LSM is used for estimating the plane rotary-tilt errors. Finally, a simulation study is performed to verify the effectiveness of the proposed on-orbit calibration method.

## 2. Optical System Error Model of Star Sensor

### 2.1. Traditional Error Model of Star Sensor

**Figure 1:**
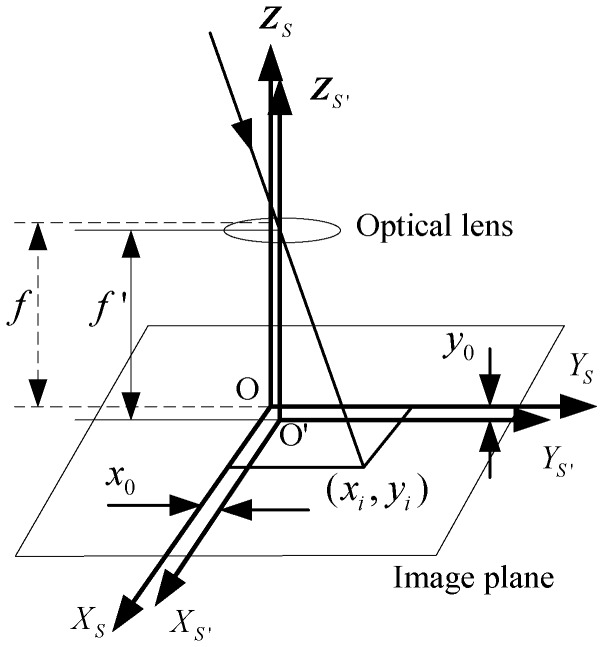
Traditional model of star sensor error.

The optical system of the star sensors is presented in Figure 1 with reference to the calibration error model of the ordinary camera in the field of photogrammetry. $OX_{S}Y_{S}Z_{S}$ denotes the star sensor coordinate system without the image plane displacement error. $O^{\prime }X_{S}^{\prime }Y_{S}^{\prime }Z_{S}^{\prime }$ denotes the star sensor coordinate system with the image plane displacement. Δx,Δy denote the principal point drifts in X and Y directions, respectively. $f^{\prime }$ denotes the focal length of the optical system with the focal length error. Δf denotes the difference between $f^{\prime }$ and f. Note that, in the traditional error model, the image plane displacement errors only include the principal point drift and the focus length error.

For the i-th star image point $\left(x_{S_{i}^{\prime }},y_{S_{i}^{\prime }}\right)$ in the image plane, its corresponding target star is $S_{i}$. The starlight vector of $S_{i}$ in the inertial coordinate system *I* can be expressed as:

(1) $$V_{i}={\left(\begin{array}{ccc} v_{i1} & v_{i2} & v_{i3} \end{array}\right)}^{T}={\left(\begin{array}{ccc} \cos\alpha_{i}\cos\beta_{i} & \sin\alpha_{i}\cos\beta_{i} & \sin\beta_{i} \end{array}\right)}^{T}$$

where*I* $\alpha_{i}$, $\beta_{i}$ are the right ascension and declination of $S_{i}$ in the celestial coordinate system, they are stored in the space-borne star map. According to the pinhole imaging model, the relation between $V_{i}$ and $\left(x_{S_{i}^{\prime }},y_{S_{i}^{\prime }}\right)$ is:

(2) $$V_{i}=C_{IO\prime }\cdot \frac{1}{\sqrt{{\left(x_{S_{i}^{\prime }}-\Delta x\right)}^{2}+{\left(y_{S_{i}^{\prime }}-\Delta y\right)}^{2}+{\left(f-\Delta f\right)}^{2}}}\left[\begin{array}{c} -\left(x_{S_{i}^{\prime }}-\Delta x\right)\\ -\left(y_{S_{i}^{\prime }}-\Delta y\right)\\ f-\Delta f \end{array}\right]$$

where, $C_{IO\prime }$ is the attitude transformation matrix of $O^{\prime }X_{S\prime }Y_{S\prime }Z_{S\prime }$ relative to ***I***. Equation (2) is the traditional error model for star sensor calibration.

The effects of the image plane tilt error, rotary error and distortions are neglected in the above model. However, with the demands for the precision of star sensors being higher and higher, the above model is no longer suitable for application in the aerospace field, so it is desirable to investigate a new error model for star sensors to solve the aforementioned problem. 

### 2.2. The Star Sensor Error Model With the Tilt and Rotation of Image Plane

**Figure 2:**
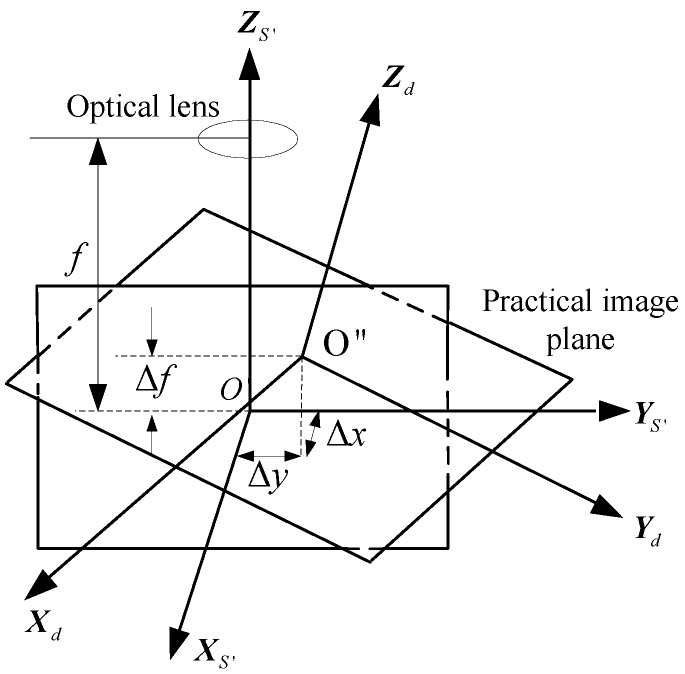
The schematic diagram of the image plane displacement error.

The temperature differences in orbit will generate displacement errors and distortions of the optical system. The displacement errors mainly include the principal point drift, rotation and tilt. The schematic diagram of the image plane displacement errors is shown in Figure 2. $O\prime \prime X_{d}Y_{d}Z_{d}$ denotes the star sensor coordinate system with the main point drift, the focal length error and the image plane tilt-rotary error, and $\left(x_{d_{i}},y_{d_{i}}\right)$ denotes the i-th star image point in the plane $O\prime \prime X_{d}Y_{d}Z_{d}$.

The rotation angles of $O\prime \prime X_{d}Y_{d}Z_{d}$ relative to $O\prime X_{S\prime }Y_{S\prime }Z_{S\prime }$ are successively ψ,θ and φ, ψ,θ and φ denote the image-plane rotary and tilt angles of the star sensor optical system. $C_{O\prime O\prime \prime }$ denotes the corresponding attitude transformation matrix:

$$C_{O\prime O\prime \prime }=\left[\begin{array}{ccc} \cos\theta \cos\varphi -\sin\theta \sin\psi \sin\varphi & \cos\varphi \sin\psi +\sin\theta \sin\varphi \cos\psi & -\cos\theta \sin\varphi \\ -\cos\theta \sin\psi & \cos\theta \cos\psi & \sin\theta \\ \sin\varphi \cos\psi +\sin\theta \cos\varphi \sin\psi & \sin\varphi \sin\psi -\sin\theta \cos\varphi \cos\psi & \cos\theta \cos\varphi \end{array}\right]$$

The star sensor error model with the main point drift, the focal length error and the image plane tilt-rotary errors is as follows:

(3) $$V_{i}=C_{IO\prime }\cdot C_{O\prime O\prime \prime }\cdot \frac{1}{\sqrt{{\left(x_{d_{i}}-\Delta x\right)}^{2}+{\left(y_{d_{i}}-\Delta y\right)}^{2}+{\left(f-\Delta f\right)}^{2}}}\left[\begin{array}{c} -\left(x_{d_{i}}-\Delta x\right)\\ -\left(y_{d_{i}}-\Delta y\right)\\ f-\Delta f \end{array}\right]$$

Let:

$$C_{IO\prime }=\left[\begin{array}{ccc} a_{11} & a_{12} & a_{12}\\ a_{21} & a_{22} & a_{23}\\ a_{31} & a_{32} & a_{33} \end{array}\right],\;C_{O\prime O\prime \prime }=\left[\begin{array}{ccc} b_{11} & b_{12} & b_{13}\\ b_{21} & b_{22} & b_{23}\\ b_{31} & b_{32} & b_{33} \end{array}\right],\;C_{O\prime O\prime \prime }^{T}\cdot C_{IO\prime }^{T}=\left[\begin{array}{ccc} c_{11} & c_{12} & c_{13}\\ c_{21} & c_{22} & c_{23}\\ c_{31} & c_{32} & c_{33} \end{array}\right].$$

and ψ,θ and φ are related to $c_{11},c_{12},c_{13},c_{21},c_{22},c_{23},c_{31},c_{32},c_{33}$.

### 2.3. The Error Model with Lens Distortions

**Figure 3:**
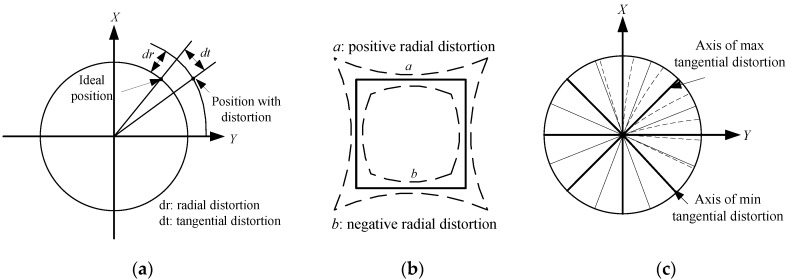
(**a**) Radial and tangential distortions; (**b**) Effect of radial distortion; (**c**) Effect of tangential distortion.

Lens distortions are also one of the main measurement errors of star sensors. The common distortion model is the Brown-Conrady model proposed by Brown in 1966 [15]. As shown in Figure 3, the model is divided into the radial distortion and the tangential distortion [27].

Mathematical expressions of the radial distortion are:

(4) $$\begin{array}{l} \delta_{x_{r}}=\lambda_{1}^{\prime }x\left(x^{2}+y^{2}\right)+O_{r}[{\left(x,y\right)}^{2}]\\ \delta_{y_{r}}=\lambda_{1}^{\prime }y\left(x^{2}+y^{2}\right)+O_{r}[{\left(x,y\right)}^{2}] \end{array}$$

$\lambda_{1}^{\prime }$ denotes the distortion coefficient, (x,y) denote the ideal position of the point, and $O_{r}[{\left(x,y\right)}^{2}]$ is the high order term of radial distortion.

The mathematical model of the tangential distortion is:

(5) $$\begin{array}{l} \delta_{x_{t}}=\mu_{1}^{\prime }\left(3x^{2}+y^{2}\right)+2\mu_{2}^{\prime }xy+O_{t}[{\left(x,y\right)}^{4}]\\ \delta_{y_{t}}=2\mu_{1}^{\prime }xy+\mu_{2}^{\prime }\left(x^{2}+3y^{2}\right)+O_{t}[{\left(x,y\right)}^{4}] \end{array}$$

$\mu_{1}^{\prime }$ and $\mu_{2}^{\prime }$ denote the tangential distortion coefficients, respectively, $O_{t}[{\left(x,y\right)}^{2}]$ denotes the high order term of the tangential distortions. Ignoring the high order terms and combining Equations (4) and (5) the distortion model can be obtained as follows:

(6) $$\begin{array}{l} \delta_{x}\left(x,y\right)=3\mu_{1}^{\prime }x^{2}+2\mu_{2}^{\prime }xy+\mu_{1}^{\prime }y^{2}+\lambda_{1}^{\prime }x\left(x^{2}+y^{2}\right)\\ \delta_{y}\left(x,y\right)=\mu_{2}^{\prime }x^{2}+2\mu_{1}^{\prime }xy+3\mu_{2}^{\prime }y^{2}+\lambda_{1}^{\prime }y\left(x^{2}+y^{2}\right) \end{array}$$

The relationships between the imaging points with the distortions and without the distortion can be expressed as:

(7) $$\begin{array}{l} x_{d}=x_{d}^{\prime }-\delta_{x_{d}}\left(x_{d},y_{d}\right)\\ y_{d}=y_{d}^{\prime }-\delta_{y_{d}}\left(x_{d},y_{d}\right) \end{array}$$

$\left(x_{d}^{\prime }，y_{d}^{\prime }\right)$ denote the actual measurement coordinates of the star image point, and $\left(x_{d},y_{d}\right)$ denote the coordinates without the optical system distortions. $\delta_{x}\left(x,y\right)$ and $\delta_{y}\left(x,y\right)$ denote non-observable coordinates of the star imaging point with (Δx,Δy), Δf, ψ, θ, and φ. Herein, the following assumptions are needed: (1) the image plane without the lens distortion is similar to the plane with the lens distortions; (2) $\delta_{x}\left(x,y\right)$ and $\delta_{y}\left(x,y\right)$ can be estimated by $\left({\hat{x}}_{d},{\hat{y}}_{d}\right)$ respectively, so the star imaging point measurement model with the lens distortions in Equation (7) can be rewritten as:

(8) $$\begin{array}{l} x_{d}=x_{d}^{\prime }-{\hat{\delta }}_{{\hat{x}}_{d}}\left({\hat{x}}_{d},{\hat{y}}_{d}\right)\\ y_{d}=y_{d}^{\prime }-{\hat{\delta }}_{{\hat{y}}_{d}}\left({\hat{x}}_{d},{\hat{y}}_{d}\right) \end{array}$$

where:

(9) $$\begin{array}{l} {\hat{\delta }}_{{\hat{x}}_{d}}\left({\hat{x}}_{d},{\hat{y}}_{d}\right)=3\mu_{1}{\hat{x}}_{d}^{2}+2\mu_{2}{\hat{x}}_{d}{\hat{y}}_{d}+\mu_{1}{\hat{y}}_{d}^{2}+\lambda_{1}{\hat{x}}_{d}\left({\hat{x}}_{d}^{2}+{\hat{y}}_{d}^{2}\right)\\ {\hat{\delta }}_{{\hat{y}}_{d}}\left({\hat{x}}_{d},{\hat{y}}_{d}\right)=\mu_{2}{\hat{x}}_{d}^{2}+2\mu_{1}{\hat{x}}_{d}{\hat{y}}_{d}+3\mu_{2}{\hat{y}}_{d}^{2}+\lambda_{1}{\hat{y}}_{d}\left({\hat{x}}_{d}^{2}+{\hat{y}}_{d}^{2}\right) \end{array}$$

In Equations (8) and (9), $\left({\hat{x}}_{d},{\hat{y}}_{d}\right)$ and $\left({\hat{\delta }}_{{\hat{x}}_{d}},{\hat{\delta }}_{{\hat{y}}_{d}}\right)$ denote the estimated values of $\left(x_{d},y_{d}\right)$ and $\left(\delta_{x},\delta_{y}\right)$, respectively. $\lambda_{1}$, $\mu_{1}$ and $\mu_{2}$ denote the distortion coefficients to be calibrated later.

### 2.4. The Comprehensive Error Model

According to the aforementioned analysis, combining Equations (3) and (8), the following equation is obtained:

(10) $$V_{i}=C_{IO\prime }\cdot C_{O\prime O\prime \prime }\cdot \frac{1}{\sqrt{{\left(x_{d_{i}}^{\prime }-\delta_{{\hat{x}}_{d_{i}}}\left({\hat{x}}_{d_{i}},{\hat{y}}_{d_{i}}\right)-\Delta x\right)}^{2}+{\left(y_{d_{i}}^{\prime }-\delta_{{\hat{y}}_{d_{i}}}\left({\hat{x}}_{d_{i}},{\hat{y}}_{d_{i}}\right)-\Delta y\right)}^{2}+{\left(f-\Delta f\right)}^{2}}}\left[\begin{array}{c} -\left(x_{d_{i}}^{\prime }-\delta_{{\hat{x}}_{d_{i}}}\left({\hat{x}}_{d_{i}},{\hat{y}}_{d_{i}}\right)-\Delta x\right)\\ -\left(y_{d_{i}}^{\prime }-\delta_{{\hat{y}}_{d_{i}}}\left({\hat{x}}_{d_{i}},{\hat{y}}_{d_{i}}\right)-\Delta y\right)\\ f-\Delta f \end{array}\right]$$

where:

(11) $${\hat{W}}_{i}=\frac{1}{\sqrt{{\left(x_{d_{i}}^{\prime }-\delta_{{\hat{x}}_{d_{i}}}\left({\hat{x}}_{d_{i}},{\hat{y}}_{d_{i}}\right)-\Delta x\right)}^{2}+{\left(y_{d_{i}}^{\prime }-\delta_{{\hat{y}}_{d_{i}}}\left({\hat{x}}_{d_{i}},{\hat{y}}_{d_{i}}\right)-\Delta y\right)}^{2}+{\left(f-\Delta f\right)}^{2}}}\left[\begin{array}{c} -\left(x_{d_{i}}^{\prime }-\delta_{{\hat{x}}_{d_{i}}}\left({\hat{x}}_{d_{i}},{\hat{y}}_{d_{i}}\right)-\Delta x\right)\\ -\left(y_{d_{i}}^{\prime }-\delta_{{\hat{y}}_{d_{i}}}\left({\hat{x}}_{d_{i}},{\hat{y}}_{d_{i}}\right)-\Delta y\right)\\ f-\Delta f \end{array}\right]$$

$V_{i}$ denotes the corresponding starlight vector in the inertial coordinate system. ${\hat{W}}_{i}$ denotes the estimated direction vector of $\left(x_{d_{i}}^{\prime }，y_{d_{i}}^{\prime }\right)$ with lens distortions in $O\prime \prime X_{d}Y_{d}Z_{d}$. $\left(x_{d_{i}}^{\prime }，y_{d_{i}}^{\prime }\right)$ are the star image points in $O\prime \prime X_{d}Y_{d}Z_{d}$.

According to Equations (9) and (10), we obtain Equation (12):

(12) $$\begin{array}{l} x_{d_{i}}^{\prime }=-\left(f-\Delta f\right)\cdot \frac{c_{11}v_{i1}+c_{12}v_{i2}+c_{13}v_{i3}}{c_{31}v_{i1}+c_{32}v_{i2}+c_{33}v_{i3}}+3\mu_{1}{\hat{x}}_{d_{i}}^{2}+2\mu_{2}{\hat{x}}_{d_{i}}{\hat{y}}_{d_{i}}^{2}+\mu_{1}{\hat{y}}_{d_{i}}^{2}+\lambda_{1}{\hat{x}}_{d_{i}}\left({\hat{x}}_{d_{i}}^{2}+{\hat{y}}_{d_{i}}^{2}\right)+\Delta x\\ y_{d_{i}}^{\prime }=-\left(f-\Delta f\right)\cdot \frac{c_{21}v_{i1}+c_{22}v_{i2}+c_{23}v_{i3}}{c_{31}v_{i1}+c_{32}v_{i2}+c_{33}v_{i3}}+\mu_{2}{\hat{x}}_{d_{i}}^{2}+2\mu_{1}{\hat{x}}_{d_{i}}{\hat{y}}_{d_{i}}+3\mu_{2}{\hat{y}}_{d_{i}}^{2}+\lambda_{1}{\hat{y}}_{d_{i}}\left({\hat{x}}_{d_{i}}^{2}+{\hat{y}}_{d_{i}}^{2}\right)+\Delta y \end{array}$$

Equation (12) is the comprehensive error model of star sensor, which is related to $\Delta x,\Delta y,\Delta f,\lambda_{1},\mu_{1},\mu_{2},$ $c_{11},c_{12},c_{13},c_{21},c_{22},c_{23},c_{31},c_{32},c_{33}$.

## 3. On-Orbit Calibration Based on a Two-Step Calibration Method

**Figure 4:**
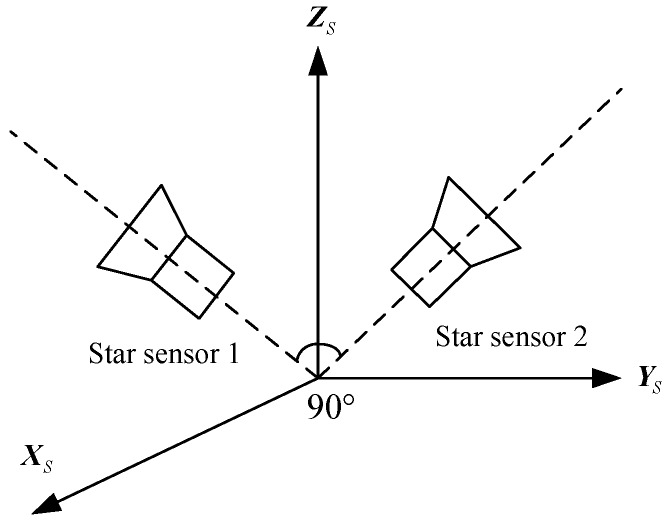
Installation configurations of double star sensors.

Generally, star sensors are the attitude sensors with the highest precision. To ensure their high accuracy, it is necessary to calibrate them on-orbit. However, the accuracy of star sensors will be limited, if they are calibrated by other attitude sensors, such as gyros. That is, the calibrated results of the star sensors are meaningless. To solve the above problem, a configuration with double star sensors is proposed in this paper, as shown in Figure 4. The installation configuration of the star sensors is fixed. The double-star sensors provide the attitude benchmark for each other, provided that they are not disabled simultaneously.

Taking advantage of the above configuration, an improved two-step calibration method based on EKF and Least Square Method (LSM) is proposed. The designed method is employed to estimate the corresponding error parameters step by step. In the first step, the EKF is used to calibrate $\Delta x,\Delta y,\Delta f,\lambda_{1},\mu_{1},\mu_{2}$.

For the non-collinear vectors ${\hat{W}}_{i}$, ${\hat{W}}_{j}$ in the star sensor coordinate system, their corresponding vectors in the inertial coordinate system are $V_{i}$, $V_{j}$. The expressions of $V_{i}$, $V_{j}$ and ${\hat{W}}_{i}$, ${\hat{W}}_{j}$ are given respectively as Equations (1) and (11). According to the double vector attitude principle [28], the attitude matrix of $O\prime \prime X_{d}Y_{d}Z_{d}$ relative to ***I*** can be obtained as Equation (13):

(13) $$C_{IO\prime \prime }=\left[\begin{array}{ccc} V_{i} & V_{j} & V_{i}\times V_{j} \end{array}\right]\cdot {\left[\begin{array}{ccc} {\hat{W}}_{i} & {\hat{W}}_{j} & {\hat{W}}_{i}\times {\hat{W}}_{j} \end{array}\right]}^{T}$$

And among these vectors, there is the following relationship:

(14) $$\cos\gamma_{ij}={\hat{W}}_{i}^{T}{\hat{W}}_{j}=V_{i}^{T}V_{j}$$

where $\gamma_{ij}$ is the angle between ${\hat{W}}_{i}$ and ${\hat{W}}_{j}$.

According to Equations (11) and (14), we have:

(15) $$V_{i}^{T}V_{j}=G_{ij}\left(\Delta x,\Delta y,\Delta f,\mu_{1},\mu_{2},\lambda_{1}\right)$$

In Equation (15), $G_{ij}\left(\Delta x,\Delta y,\Delta f,\mu_{1},\mu_{2},\lambda_{1}\right)$ is a function of $\left(\Delta x,\Delta y,\Delta f,\mu_{1},\mu_{2},\lambda_{1}\right)$ and $\lambda_{1}$ $\left(\Delta \hat{x},\Delta \hat{y},\Delta \hat{f},{\hat{\mu }}_{1},{\hat{\mu }}_{2},{\hat{\lambda }}_{1}\right)$ denote the estimated values of $\left(\Delta x,\Delta y,\Delta f,\mu_{1},\mu_{2},\lambda_{1}\right)\prime$, $\left(\delta x,\delta y,\delta f,\Delta \mu_{1},\Delta \mu_{2},\Delta \lambda_{1}\right)$ denote the difference between the estimated value and the nominal value. Let $X=\left(\Delta x,\Delta y,\Delta f,\mu_{1},\mu_{2},\lambda_{1}\right)\prime$, $\hat{X}=\left(\Delta \hat{x},\Delta \hat{y},\Delta \hat{f},{\hat{\mu }}_{1},{\hat{\mu }}_{2},{\hat{\lambda }}_{1}\right)\prime$, $\xi =\left(\delta x,\delta y,\delta f,\Delta \mu_{1},\Delta \mu_{2},\Delta \lambda_{1}\right)\prime$. $X,\hat{X}$ and ξ are respectively the nominal value, the estimated value and the difference, the relational expression is:

(16) $$X=\hat{X}+\xi$$

Based on the star sensor error model, the system functions of the EKF are established as:

(17) $$\{\begin{array}{c} \xi \left(k+1\right)=f\left(k,\xi \left(k\right)\right)+w\left(k\right)\\ y(k)=h\left(k,\xi \left(k\right)\right)+v\left(k\right) \end{array}$$

where:

(18) f(k,ξ(k))=ξ(k)

(19) $$h(k,\xi (k))=\left[\begin{array}{c} {\left({\hat{W}}_{1}^{k}\right)}^{T}{\hat{W}}_{2}^{k}-{\left(V_{1}^{k}\right)}^{T}V_{2}^{k}\\ \cdots \\ {\left({\hat{W}}_{1}^{k}\right)}^{T}{\hat{W}}_{n}^{k}-{\left(V_{1}^{k}\right)}^{T}V_{n}^{k}\\ {\left({\hat{W}}_{2}^{k}\right)}^{T}{\hat{W}}_{3}^{k}-{\left(V_{2}^{k}\right)}^{T}V_{3}^{k}\\ \cdots \\ {\left({\hat{W}}_{n-1}^{k}\right)}^{T}{\hat{W}}_{n}^{k}-{\left(V_{n-1}^{k}\right)}^{T}V_{n}^{k} \end{array}\right]$$

In Equation (19), n denotes the number of star imaging points. h(k) denotes the difference between the real angular distances and the calibrated angular distances after k times of iteration. w(k) denotes the systematic noise, and v(k) denotes the measured noise. w(k) and v(k) are the Gaussian White noise with zero mean [7,18,20,21]. They conform to the following rules.

(20) $$E\left[w\left(k\right)w{\left(k\right)}^{\text{T}}\right]=Q^{w}\left(k\right),E\left[v\left(k\right)\right]=0,\;E\left[v\left(k\right)v{\left(k\right)}^{\text{T}}\right]=Q^{v}\left(k\right),\text{E}\left[w\left(k\right)v{\left(k\right)}^{\text{T}}\right]=0,E\left[w\left(k\right)\right]=0$$

The state variables can be described as:

(21) $$\hat{\xi }\left(k+1\right)=\hat{\xi }\left(k\right)+N\left(k\right)\left[y\left(k\right)-h\left(k,\hat{\xi }\left(k\right)\right)\right]$$

$\hat{\xi }(k)$ denotes the estimated value of ξ(k). The processes of measurement update and time update are expressed respectively as:

(22) $$K\left(k\right)=F\left(k,\hat{\xi }\left(k\right)\right)P\left(k\right)H^{\text{T}}\left(k,\hat{\xi }\left(k\right)\right)\times {\left[H\left(k,\hat{\xi }\left(k\right)\right)P\left(k\right)H^{\text{T}}\left(k,\hat{\xi }\left(k\right)\right)+Q^{v}\left(k\right)\right]}^{-1}$$

(23) $$P\left(k+1\right)=F\left(k,\hat{\xi }\left(k\right)\right)P\left(k\right)F^{\text{T}}\left(k,\hat{\xi }\left(k\right)\right)+Q^{w}\left(k\right)-K\left(k\right)\left[Q^{v}\left(k\right)+H\left(k,\hat{\xi }\left(k\right)\right)P\left(k\right)H^{\text{T}}\left(k,\hat{\xi }\left(k\right)\right)\right]K^{\text{T}}\left(k\right)$$

(24) $$X\left(k+1\right)=X\left(k\right)+K\left(k\right)\left[\hat{\xi }\left(k+1\right)-Q^{v}\left(k\right)X\left(k\right)\right]$$

where:

(25) $$F\left(k,\hat{\xi }\right)={\frac{\partial }{\partial \xi }f\left(k,\hat{\xi }\right)|}_{\xi =\hat{\xi }}=I$$

(26) $$H\left(k,\hat{\xi }\right)={\frac{\partial }{\partial \xi }h\left(k,\hat{\xi }\right)|}_{\xi =\hat{\xi }}=\left[\begin{array}{ccc} \frac{\partial }{\partial \left(\Delta x\right)}{{\hat{W}}_{1}}^{\text{T}}{\hat{W}}_{2} & \cdots & \frac{\partial }{\partial \lambda_{1}}{{\hat{W}}_{1}}^{\text{T}}{\hat{W}}_{2}\\ \vdots & \cdots & \vdots \\ \frac{\partial }{\partial \left(\Delta x\right)}{{\hat{W}}_{n-1}}^{\text{T}}{\hat{W}}_{n} & \cdots & \frac{\partial }{\partial \lambda_{1}}{{\hat{W}}_{n-1}}^{\text{T}}{\hat{W}}_{n} \end{array}\right]$$

**Figure 5:**
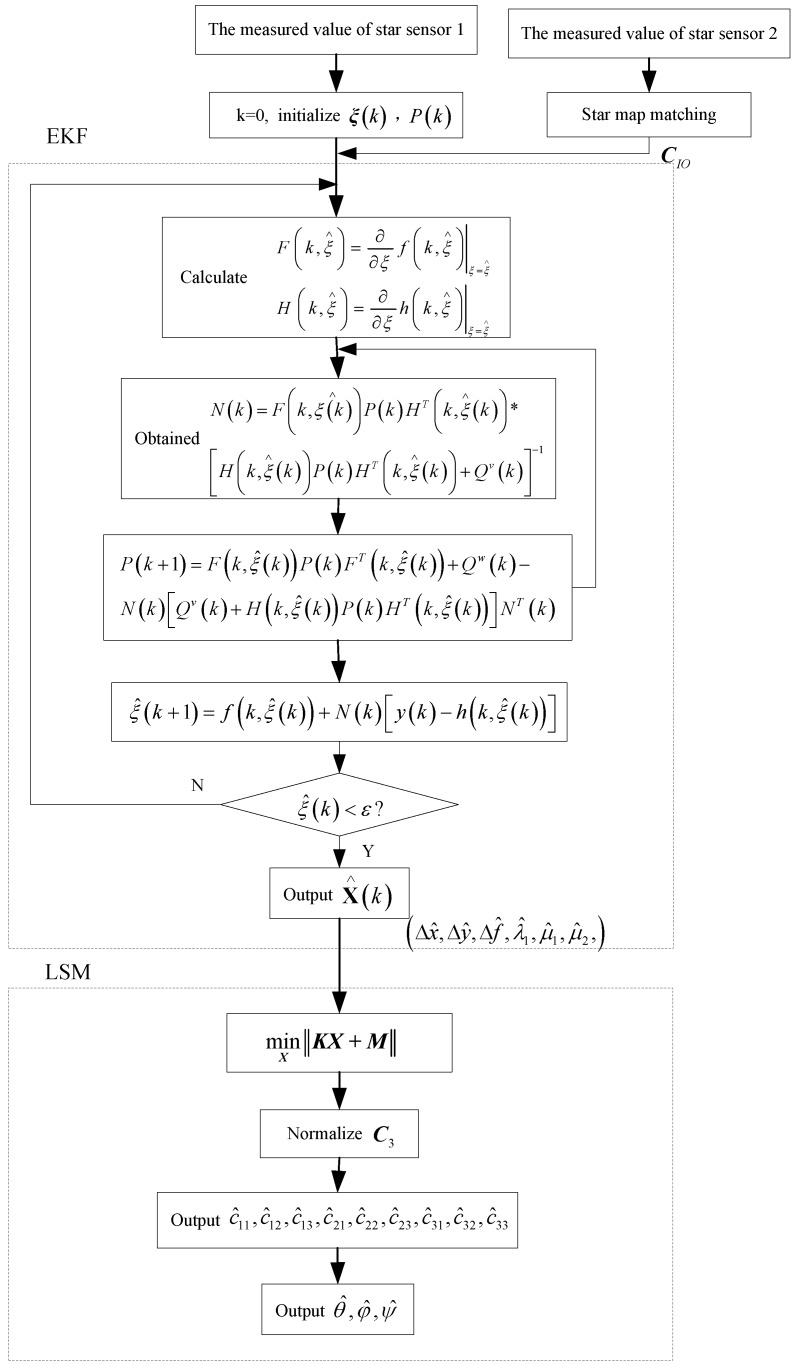
Flow chart of on-orbit calibration of double star sensors.

In Equations (25) and (26), $F\left(k,\hat{\xi }\right)$ and $H\left(k,\hat{\xi }\right)$ respectively denote the Jacobi matrix of $f\left(k,\hat{\xi }\right)$ and $h\left(k,\hat{\xi }\right)$, K(k) denotes the EKF gain, P(k) denotes the forecast variance matrix, $Q^{w}\left(k\right)$ and $Q^{v}\left(k\right)$ are respectively the systematic noise matrix and the measured noise matrix. The flow chart of the EKF is shown in the first dotted box of Figure 5.

The estimated results from the first step are substituted in Equation (12), and then the matrix Equation (28) is available:

(27) $$\begin{array}{l} \left(x_{d_{i}}^{\prime }-\delta_{{\hat{x}}_{d_{i}}}\left({\hat{x}}_{d_{i}},{\hat{y}}_{d_{i}}\right)-\Delta x\right)v_{i1}c_{31}+\left(x_{d_{i}}^{\prime }-\delta_{{\hat{x}}_{d_{i}}}\left({\hat{x}}_{d_{i}},{\hat{y}}_{d_{i}}\right)-\Delta x\right)v_{i2}c_{32}+\left(x_{d_{i}}^{\prime }-\delta_{{\hat{x}}_{d_{i}}}\left({\hat{x}}_{d_{i}},{\hat{y}}_{d_{i}}\right)-\Delta x\right)v_{i3}c_{33}-\\ \hat{f}\prime v_{i1}c_{11}-\hat{f}\prime v_{i2}c_{12}-\hat{f}\prime v_{i3}c_{13}=0\\ \left(y_{d_{i}}^{\prime }-\delta_{{\hat{y}}_{d_{i}}}\left({\hat{x}}_{d_{i}},{\hat{y}}_{d_{i}}\right)-\Delta y\right)v_{i1}c_{31}+\left(y_{d_{i}}^{\prime }-\delta_{{\hat{y}}_{d_{i}}}\left({\hat{x}}_{d_{i}},{\hat{y}}_{d_{i}}\right)-\Delta y\right)v_{i2}c_{32}+\left(y_{d_{i}}^{\prime }-\delta_{{\hat{y}}_{d_{i}}}\left({\hat{x}}_{d_{i}},{\hat{y}}_{d_{i}}\right)-\Delta y\right)v_{i3}c_{33}-\\ \hat{f}\prime v_{i1}c_{21}-\hat{f}\prime v_{i2}c_{22}-\hat{f}\prime v_{i3}c_{23}=0 \end{array}$$

(28) $$\left(\begin{array}{ccc} -V_{1} & 0 & \left(x_{d_{1}}^{\prime }-\delta_{{\hat{x}}_{d_{1}}}\left({\hat{x}}_{d_{1}},{\hat{y}}_{d_{1}}\right)-\Delta \hat{x}\right)\cdot V_{1}\\ 0 & -V_{1} & \left(y_{d_{1}}^{\prime }-\delta_{{\hat{y}}_{d_{1}}}\left({\hat{x}}_{d_{1}},{\hat{y}}_{d_{1}}\right)-\Delta \hat{y}\right)\cdot V_{1}\\ \cdot \cdot \cdot & \cdot \cdot \cdot & \cdot \cdot \cdot \\ -V_{n} & 0 & \left(x_{d_{n}}^{\prime }-\delta_{{\hat{x}}_{d_{n}}}\left({\hat{x}}_{d_{n}},{\hat{y}}_{d_{n}}\right)-\Delta \hat{x}\right)\cdot V_{n}\\ 0 & -V_{n} & \left(y_{d_{n}}^{\prime }-\delta_{{\hat{y}}_{d_{n}}}\left({\hat{x}}_{d_{n}},{\hat{y}}_{d_{n}}\right)-\Delta \hat{y}\right)\cdot V_{n} \end{array}\right)\left(\begin{array}{c} \hat{f}\cdot C_{1}\\ \hat{f}\cdot C_{2}\\ C_{3} \end{array}\right)=0$$

where: $C_{1}=\left[\begin{array}{c} c_{11}\\ c_{12}\\ c_{13} \end{array}\right]$, $C_{2}=\left[\begin{array}{c} c_{21}\\ c_{22}\\ c_{23} \end{array}\right]$ and $C_{3}=\left[\begin{array}{c} c_{31}\\ c_{32}\\ c_{33} \end{array}\right]$.

In accordance with the least square estimation, Equation (28) can be rewritten into the following nonhomogeneous linear forms:

(29) KΧ+Μ=0

where:

$$K=\left(\begin{array}{cccc} -V_{1} & 0 & \left(x_{d_{1}}^{\prime }-\delta_{{\hat{x}}_{d_{1}}}\left({\hat{x}}_{d_{1}},{\hat{y}}_{d_{1}}\right)-\Delta \hat{x}\right)\cdot v_{11} & \left(x_{d_{1}}^{\prime }-\delta_{{\hat{x}}_{d_{1}}}\left({\hat{x}}_{d_{1}},{\hat{y}}_{d_{1}}\right)-\Delta \hat{x}\right)\cdot v_{12}\\ 0 & -V_{1} & \left(y_{d_{1}}^{\prime }-\delta_{{\hat{y}}_{d_{1}}}\left({\hat{x}}_{d_{1}},{\hat{y}}_{d_{1}}\right)-\Delta \hat{y}\right)\cdot v_{11} & \left(y_{d_{1}}^{\prime }-\delta_{{\hat{y}}_{d_{1}}}\left({\hat{x}}_{d_{1}},{\hat{y}}_{d_{1}}\right)-\Delta \hat{y}\right)\cdot v_{12}\\ \cdot \cdot \cdot & \cdot \cdot \cdot & \cdot \cdot \cdot & \cdot \cdot \cdot \\ -V_{n} & 0 & \left(x_{d_{n}}^{\prime }-\delta_{{\hat{x}}_{d_{n}}}\left({\hat{x}}_{d_{n}},{\hat{y}}_{d_{n}}\right)-\Delta \hat{x}\right)\cdot v_{n1} & \left(x_{d_{n}}^{\prime }-\delta_{{\hat{x}}_{d_{n}}}\left({\hat{x}}_{d_{n}},{\hat{y}}_{d_{n}}\right)-\Delta \hat{x}\right)\cdot v_{n2}\\ 0 & -V_{n} & \left(y_{d_{n}}^{\prime }-\delta_{{\hat{y}}_{d_{n}}}\left({\hat{x}}_{d_{n}},{\hat{y}}_{d_{n}}\right)-\Delta \hat{y}\right)\cdot v_{n1} & \left(y_{d_{n}}^{\prime }-\delta_{{\hat{y}}_{d_{n}}}\left({\hat{x}}_{d_{n}},{\hat{y}}_{d_{n}}\right)-\Delta \hat{y}\right)\cdot v_{n2} \end{array}\right),\;X=\left[\begin{array}{c} X_{1}\\ X_{2}\\ X_{3} \end{array}\right]=\left(\begin{array}{c} \hat{f}\cdot C_{1}\\ \hat{f}\cdot C_{2}\\ \left(\begin{array}{c} c_{31}\\ c_{32} \end{array}\right) \end{array}\right),$$

$$M=\left(\begin{array}{c} \left(x_{d_{1}}^{\prime }-\delta_{{\hat{x}}_{d_{1}}}\left({\hat{x}}_{d_{1}},{\hat{y}}_{d_{1}}\right)-\Delta \hat{x}\right)\cdot v_{13}c_{33}\\ \left(y_{d_{1}}^{\prime }-\delta_{{\hat{y}}_{d_{1}}}\left({\hat{x}}_{d_{1}},{\hat{y}}_{d_{1}}\right)-\Delta \hat{y}\right)\cdot v_{13}c_{33}\\ \cdot \cdot \cdot \\ \left(x_{d_{n}}^{\prime }-\delta_{{\hat{x}}_{d_{n}}}\left({\hat{x}}_{d_{n}},{\hat{y}}_{d_{n}}\right)-\Delta \hat{x}\right)\cdot v_{n3}c_{33}\\ \left(y_{d_{n}}^{\prime }-\delta_{{\hat{y}}_{d_{n}}}\left({\hat{x}}_{d_{n}},{\hat{y}}_{d_{n}}\right)-\Delta \hat{y}\right)\cdot v_{n3}c_{33} \end{array}\right)$$

Let ${\tilde{c}}_{33}=1$, ${\tilde{c}}_{3}=[{\tilde{c}}_{31}\begin{array}{cc} \begin{array}{cc} & {\tilde{c}}_{2} \end{array} & 1 \end{array}]\prime$. Based on the least square method $\underset{X}{\min\|KX+M\|}$, the estimated results ${\tilde{X}}_{1},{\tilde{X}}_{2}$ and ${\tilde{X}}_{3}$ are available:

(30) $$\begin{array}{l} {\tilde{C}}_{1}={\tilde{X}}_{1}/\hat{f}\\ {\tilde{C}}_{2}={\tilde{X}}_{2}/\hat{f}\\ {\tilde{C}}_{3}=\left[\begin{array}{cc} {\tilde{X}}_{3}\prime & 1 \end{array}\right]\prime \end{array}$$

where $\hat{f}=f+\Delta \hat{f}$. Finally, the results of the least squares estimation can be obtained from ${\tilde{C}}_{1},{\tilde{C}}_{2}$ and ${\tilde{C}}_{3}$ being orthonormalized. Now that ${\hat{c}}_{11},{\hat{c}}_{12},{\hat{c}}_{13},{\hat{c}}_{21},{\hat{c}}_{22},{\hat{c}}_{23},{\hat{c}}_{31},{\hat{c}}_{32},{\hat{c}}_{33}$ are determined, subsequently the image-plane rotary and tilt angles of the star sensor optical system $\hat{\psi },\hat{\theta },\hat{\varphi }$ are calculated.

## 4. Simulation and Analysis

In this section, a simulation is provided to demonstrate the effectiveness of the proposed on-orbit calibration method for star sensors. Firstly, in order to analyze the effect of the optical system errors on the measurement accuracy of the star imaging points quantitatively, the specific parameters of star sensors are shown in Table 1.

**Table 1:** The parameters of the star sensors.

Focal Length *f*	45 mm
Field of view *ω*	10° × 10°
Pixels	25 μm × 25 μm
The main point drift (Δ*x*, Δ*y*)/(pixel)	(3.75, −3)
The focal length error Δ*f*	1.58 mm
Tangential distortion coefficient *μ*_1_	0.15
Tangential distortion coefficient *μ*_2_	−0.2
Tangential distortion coefficient *λ*_1_	0.03
The image-plane rotary error angle *ψ*	0.2°
The image-plane tilt error angle *θ*	0.4°
The image-plane tilt error angle *φ*	−0.15

**Figure 6:**
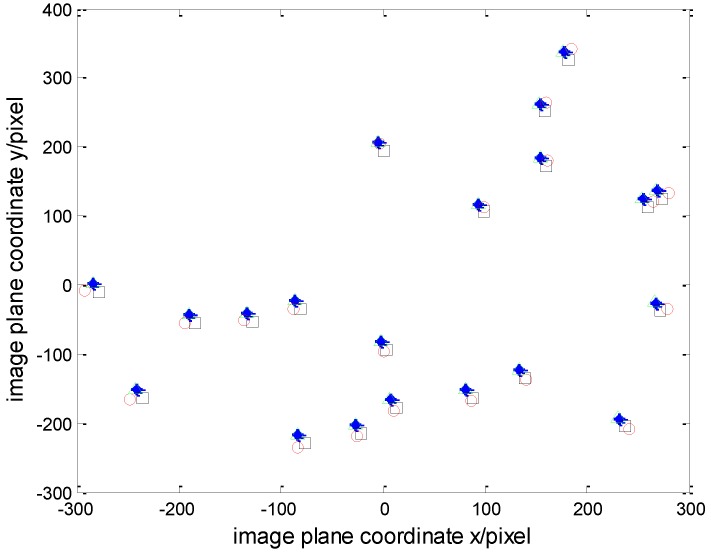
The measured values and theoretical values of the coordinates of star image points.

Twenty groups of star image points are used in the simulation, and the corresponding coordinates are shown in Figure 6 according to the error models described in Section 2. In Figure 6, the symbol “○” denotes the coordinates $\left(x_{i},y_{i}\right)$ of star image points without the main point drift, the focal length error, the image plan rotary-tilt errors, or the distortions; the symbol “□” denotes the coordinates $\left(x_{z_{i}},y_{z_{i}}\right)$ of star image points with the main point drift, the focal length error and without the image plan rotary-tilt errors or the distortion; the symbol “△” denotes the coordinates $\left(x_{s_{i}},y_{s_{i}}\right)$ of star image points with the main point drift, the focal length error, the image plan rotary-tilt errors and without the distortion; the symbol “*” denotes the star image points’ coordinates $\left(x_{d_{i}},y_{d_{i}}\right)$ of star image points containing various optical system errors. Then the coordinates of star image points are given in Table 2.

Analyzing the coordinates of star image points in Table 2, the impact of the main point drift, the focal length error, the image plane rotary error, the image plane tilt error and the distortions on the position accuracy of the star image points can be indicated by 6.96%, 4.97%, 12.37%, 5.38% and 15.42%, respectively. Therefore, in addition to the main point drift and the focal length error, small image-plane rotary-tile angles and the distortions have a great effect on the accuracy of the star sensors.

**Table 2:** The coordinates of star image points.

	$\left(x_{i},y_{i}\right)$/(pixel)	$\left(x_{z_{i}},y_{z_{i}}\right)$/(pixel)	$\left(x_{s_{i}},y_{s_{i}}\right)$/(pixel)	$\left(x_{d_{i}},y_{d_{i}}\right)$/(pixel)
1	(284.75, −89.03)	(294.76, −97.94)	(282.79, −85.47)	(290.04, −99.57)
2	(−13.05, −250.52)	(−17.05, −253.73)	(−15.99, −237.92)	(−12.33, −258.19)
3	(181.172, 349.09)	(194.81, 324.83)	(168.78, 338.25)	(180.30, 325.14)
4	(−74.81, −121.78)	(−52.18, −129.51)	(−83.76, −113.13)	(−83.24, −116.56)
5	(−20.85, 109.69)	(−11.53, 93.84)	(−28.99, 109.71)	(−26.99, 106.38)
6	(143.1128, −46.60)	(158.09, −56.96)	(127.23, −43.06)	(133.22, −45.89)
7	(64.65, −231.27)	(82.38, −235.15)	(49.47, −220.03)	(57.01, −239.53)
8	(−148.62, 35.11)	(−123.41, 21.88)	(−153.40, 39.22)	(−147.59, 36.21)
9	(−133.92, −222.14)	(−109.21, −226.34)	(−141.99, −209.31)	(−142.55, −223.80)
10	(45.78, 235.53)	(64.18, 215.26)	(36.83, 230.44)	(40.45, 219.96)
11	(116.44, 24.70)	(132.35, 11.83)	(102.43, 25.96)	(105.06, 25.06)
12	(117.06, −46.35)	(132.95, −56.73)	(102.16, −42.52)	(106.30, −44.52)
13	(−56.94, −160.53)	(−34.94, −166.89)	(−66.94, −150.69)	(−66.41, −157.32)
14	(−255.16, 45.09)	(−226.20, 31.50)	(−256.53, 50.17)	(−244.04, 42.37)
15	(−203.55, 90.41)	(−176.40, 75.23)	(−206.00, 93.39)	(−194.84, 85.53)
16	(−140.52, −326.01)	(−115.58, −326.56)	(−149.51, −309.36)	(−151.68, −344.38)
17	(−199.04, −142.54)	(−172.05, −149.53)	(−204.13, −131.85)	(−202.20, −139.23)
18	(117.72, 310.05)	(133.59, 287.16)	(107.14, 301.46)	(113.91, 287.93)
19	(−183.86, −145.84)	(−157.40, −152.72)	(−189.47, −135.20)	(−187.95, −142.31)
20	(−338.88, 94.97)	(−306.98, 79.63)	(−337.32, 99.53)	(−316.89, 84.39)

**Figure 7:**
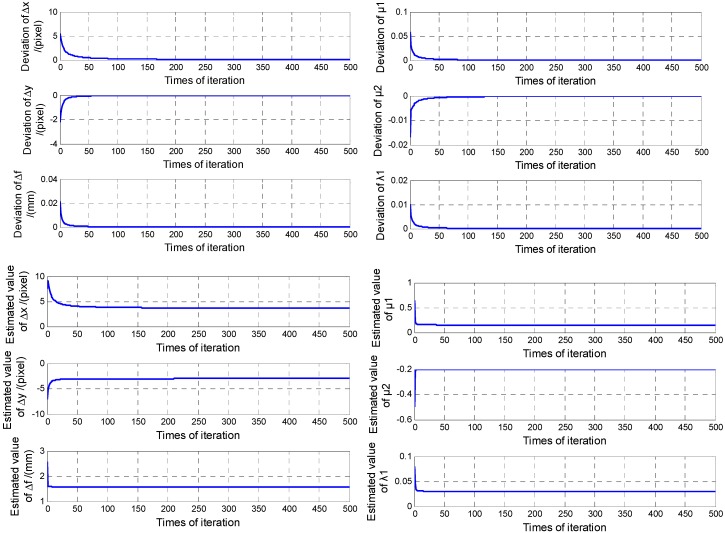
On-orbit calibration results of EKF.

On the basis of the two-step method proposed in Section 3, the image plane errors Δx,Δy,Δf, $\mu_{1},\mu_{2},\lambda_{1}$ can be estimated first by the EKF. The measured values are 20-group coordinates of stars image points. The centroid noise is considered as a random Gaussian noise with zero mean and non-zero standard deviation. Generally, it ranges from 0 to 0.5 pixels. In this simulation, the mean and standard deviation of the noise are respectively 0 and 0.04 pixel. The results of the EKF are shown in Figure 7.

Using the EKF, the calibrated results are $\Delta \hat{x}=3.656pixel$, $\Delta \hat{y}=-3.032pixel$, $\Delta \hat{f}=1.5806mm$, ${\hat{\mu }}_{1}=0.1497$, ${\hat{\mu }}_{2}=-0.1996$ and ${\hat{\lambda }}_{1}=0.0300$. The estimated errors are 1.53%, 0.96%, 0.038%, 0.02%, 0.02% and 0, respectively. Further, using the LSM, the estimated values of ${\hat{c}}_{11},{\hat{c}}_{12},{\hat{c}}_{13},{\hat{c}}_{21},{\hat{c}}_{22},$ ${\hat{c}}_{23},{\hat{c}}_{31},{\hat{c}}_{32}$ and ${\hat{c}}_{33}$ can be available, they are provided in Table 3.

**Table 3:** Calibration results of LSM.

Estimated Parameters	Theoretical Values	LSM Results
${\hat{c}}_{11}$	−0.57474	−0.57458
${\hat{c}}_{12}$	−0.75883	−0.75895
${\hat{c}}_{13}$	−0.30638	−0.30635
${\hat{c}}_{21}$	−0.12095	−0.12111
${\hat{c}}_{22}$	−0.29149	−0.29126
${\hat{c}}_{23}$	0.94890	0.94898
${\hat{c}}_{31}$	−0.80937	−0.81074
${\hat{c}}_{32}$	0.58242	0.58259
${\hat{c}}_{33}$	0.07570	−0.07586

Furthermore, the rotary-tilt angles of the star sensor image plane as shown in Table 4 are calculated by using the values in Table 3, and the ultimate results of LSM are ψ=0.19900°, θ=0.39914° and φ=−0.15057°. The errors are Δψ=3.60″, Δθ=3.096″ and Δφ=2.052″, and the accuracies of the calibration are respectively 0.5%, 0.22% and 0.38%.

**Table 4:** Estimation results of the image-plane rotary-tilt angles.

Estimated Parameters	Theoretical Values	Estimated Results of LSM
$\hat{\psi }$	0.2°	0.19900°
$\hat{\theta }$	0.4	0.39914
$\hat{\varphi }$ **	−0.15	−0.15057°

The simulation results indicate that the estimated values are stabilized rapidly around the expected values after 30–40 rounds of iteration. The response speed of the improved two-step calibration method is fast, and the maximum error is 1.53%. The image-plane rotary-tilt angles can be available with LSM, and the maximum deviation angle is 3.60”. The position error of the star imaging points is improved from 15.42% to 1.389% after calibration. Therefore, the measurement model of the star sensor is correct, the two-step calibration method is effective, and the calibration results are satisfactory.

## 5. Conclusions

The error factors of the main point drift, the focal length error, the image plane rotary-tilt errors and the distortions have been analyzed in this paper. A novel on-orbit measurement model of star sensors which considers the image-plane rotary-tilt errors and the distortions has been explored based on the incomplete traditional measurement model. According to the characteristics of the novel model, a modified two-step calibration method has been designed to calibrate the model parameters. First, the EKF is utilized to estimate the main point drift, the focal length error, the radial distortion and the tangential distortion, as well as to effectively eliminate the impact of measurement noise. Then the LSM is applied to estimate the image plane rotary-tilt errors accurately. The improved two-step calibration method can solve the problems of many parameters needing to be calibrated in the measurement model and strong nonlinearity among these parameters. Ultimately, the simulation results have indicated that the star sensor on-orbit measurement model is accurate, and the two-step calibration method is feasible. It has been proved that the proposed model and the modified calibration method can be used to effectively improve the on-orbit measurement accuracy and the attitude accuracy of the star sensors.
